# Do Reported Effects of Acute Aerobic Exercise on Subsequent Higher Cognitive Performances Remain if Tested against an Instructed Self-Myofascial Release Training Control Group? A Randomized Controlled Trial

**DOI:** 10.1371/journal.pone.0167818

**Published:** 2016-12-08

**Authors:** Max Oberste, Wilhelm Bloch, Sven T. Hübner, Philipp Zimmer

**Affiliations:** 1 Department for molecular and cellular sports medicine, German Sports University Cologne, Am Sportpark Müngersdorf 6, Cologne, Germany; 2 National Center for Tumor Diseases (NCT) and German Cancer Research Center, Im Neuenheimer Feld 280, Heidelberg, Germany; University of Rome, ITALY

## Abstract

A substantial body of evidence suggests positive effects of acute aerobic exercise (AAE) on subsequent higher cognitive functions in healthy young adults. These effects are widely understood as a result of the ongoing physiological adaptation processes induced by the preceding AAE. However, designs of published studies do not control for placebo, Hawthorne and subject expectancy effects. Therefore, these studies do not, at a high degree of validity, allow attributing effects of AEE on subsequent cognitive performance to exercise induced physical arousal. In the present study, we applied a randomized controlled blinded experiment to provide robust evidence for a physiological basis of exercise induced cognitive facilitation. Beyond that, the dose response relationship between AAE`s intensity and subsequent cognitive performances as well as a potentially mediating role of peripheral lactate in AAE induced cognitive facilitation was investigated. The 121 healthy young subjects who participated in this study were assigned randomly into 3 exercise groups and a self-myofascial release training control group. Exercise groups comprised a low, moderate and high intensity condition in which participants cycled on an ergometer at a heart rate corresponding to 45–50%, 65–70% and 85–90% of their individual maximum heart rate, respectively, for 35 minutes. Participants assigned to the control group completed a 35 minute instructed self-massage intervention using a foam roll. Before and after treatment, participants completed computer based versions of the Stroop task and the Trail Making Test as well as a free recall task. None of the applied exercise regimes exerted a significant effect on participants`performance at any of the applied cognitive testing procedure if compared to self-myofascial release training control group. Post hoc power analyses revealed no effect in the population of f = .2 or larger at a risk of type II error (β) ≤.183 for all measured variables. Our results, therefore, indicate that AAE induced cognitive facilitation is not (exclusively) based on physiological effects. Even if there is a substantial contribution of physiological adaptations to AAE in reported AAE induced cognitive facilitation, in this study, peripheral lactate could not be confirmed as such a factor. Peripheral lactate concentrations and cognitive testing performances after exercise showed rather small empirical and no significant associations. Our results suggest that other psychosocial aspects like expectations and social attention play an important role in AAE induced cognitive facilitation.

## Introduction

Acute aerobic exercise (AAE) was reported to improve basic information processing speed [1] as well as higher cognitive functions like attention [2], memory [3] and executive functioning [4] in young healthy adults. These positive effects of AAE on subsequent cognitive processes are widely understood as a result of the ongoing physiological adaptation processes induced by the preceding acute physical exertion [1, 5–7]. Several physiological processes, on the one hand, known to be acutely affected by aerobic exercise and, on the other hand, known (or at least suspected) to positively influence CNS functioning are discussed as potential mediators of exercise induced cognitive facilitation. Amongst others, increase in cerebral blood flow [8] and improved metabolic status of cerebral neurons [7] are favored explanations for AAE induced cognitive facilitation.

However, compared to animal studies, solid evidence about these physiological processes potentially underlying AAE induced cognitive facilitation in humans is sparse. This is because techniques to directly investigate those mechanisms are hardly feasible in human studies. Invasive procedures (e.g. withdrawal of blood samples from internal jugular vein, brain biopsy, lactate infusion etc.) would be required which cannot easily be implemented. Against this backdrop, research on AAE induced cognitive facilitation on the basis of a physiological rational depends on designs that comprise exercise and control conditions fulfilling the ceteris paribus clause in terms of all other potential mediators (e.g. participants`expectations, social attention etc.) but differing in terms of participants`physiological arousal.

However, existing studies with healthy young adults are far from testing the effects of AAE on subsequent cognitive performances in relation to a control group that provokes similar expectations and shows comparable social attention. Typically, a passive control group was applied. Participants were instructed to sit on their own in a waiting room [3, 6, 7, 9, 10] or on an ergometer [11–14] for approximately the amount of time experimental group participants exercised. With these research designs, one might legitimately argue that positive effects of AAE on subsequent cognition are not due to the physiological effects of the preceding exercise but rather to exercise group participants`positive expectations of the treatment in the sense of a placebo effect [15]. Moreover, one might argue that the effects could be a result of the close supervision exercise group participants received during treatment while control group participants were left to their own devices [16].

In some studies that reported positive effects of AAE on subsequent cognition, control group participants were instructed to read or watch a video [4, 12, 17–20]. Here, control group participants received an active treatment. However, it remains questionable if reading or watching a video raises similar positive expectations of treatment induced cognitive benefits among control group participants compared to how exercise treatment does among exercise group participants. If Hawthorne effect related bias can be ruled out with these control groups seems even more questionable and one might even suspect the control group treatment (reading or watching a video) to be cognitive demanding deteriorating subsequent cognitive performances.

Murray and Russoniello [2] had control group participants monitor experimental group participants` heart rate and keep up their motivation during exercise sessions. With this approach Murray and Russoniello [2] aimed to keep control group participants` mentally active while the exercise group completed their bout. However, if this design fulfills ceteris paribus clause in terms of other potential mediators seems also highly questionable.

Moreover, existing studies on the effects of AAE on subsequent cognitive performances in healthy young adults can be considered open trials. Participants were either directly informed about researchers` hypothesis [3, 6, 7, 9, 10] or they could easily derive it from context [2, 11–14]. With these designs, it cannot be ruled out that subject expectancy effects underlie detected positive effects of AAE on subsequent cognitive performances rather than physiological adaptations to preceding exercise.

In the light of the forgoing, the primary aim of this study was to investigate the effect of AAE on subsequent cognitive performances applying a research design that, at a high degree of validity, allows attributing effects of AAE on cognition to the physiological impact of the preceding exertion. It was hypothesized that reported positive effects of AAE on subsequent cognitive performances can also be detected if AAE is tested against a placebo control treatment in a blinded experiment. This would provide robust evidence for a physiological basis of exercise induced cognitive facilitation. In the present study, an instructed self-massage intervention using a foam roll was selected as placebo control treatment. Self-massage foam roll intervention was selected as placebo control treatment for the following three reasons: (i) foam roll treatments have become a common practice in professional sports and recreational exercise [21] and therefore might raise similar positive expectations in participants compared to exercise treatment; (ii) all participants receive a comparable amount of attention by the experimenter since both interventions, foam roll treatment and cycle ergometer exercise, are closely supervised; (iii) the here applied foam roll treatment does not induce physiological arousal (controlled by heart rate and peripheral lactate measurement). A wide range of cognitive domains was tested. Computerized versions of the Stroop task, the Trail Making Test (TMT) and a free recall test were used demanding executive functioning subdomains interference control (Stroop task) and set-shifting (TMT part B) as well as selective attention (Stroop task and TMT-A) and declarative memory (free recall test).

Second aim of the present study was to investigate the dose-response relationship between AAE`s intensity and subsequent cognitive performances. The extent of the physiological adaptations to acute exercise discussed as potential mediators of subsequent cognitive facilitation [5, 17] is highly dependent on exercise`s intensity [22]. Therefore, an intensity dependent effect of AAE on subsequent cognitive performance was hypothesized. In the present study, besides placebo control group (CG), a low intensity (LI), a moderate intensity (MI) and a high intensity exercise group (HI) were realized and participants were randomly assigned to one of these four groups.

Third aim of the present study was to investigate peripheral lactate as potential mediator of exercise induced cognitive facilitation as was recently proposed by Skriver and colleagues [7]. With exhaustive cardiovascular exercise, during which large muscle groups are activated and anaerobic metabolism prevails, peripheral lactate levels increase significantly and remain elevated for some time after exercise [23]. It was shown that during such intense exercise and subsequent recovery period the brain takes up large amounts of the peripherally originated lactate [24]. Regarding the significance of lactate for CNS functioning, research has well established that not glucose but lactate is neurons´ preferred energy source in the brain [25–27]. Against this backdrop, in the present study, we looked for potential correlations between peripheral lactate concentrations and cognitive testing scores (Stroop, TMT and free recall) hypothesizing positive associations.

## Materials and Methods

### Sample size calculation

Prior to data collection, a power analysis was conducted to determine that particular sample size needed to detect an effect of defined size with a certain probability (statistical test-power: 1-β) as far as this effect really does exist in the population. Current research literature reports small to moderate positive effects of AAE on subsequent higher cognitive performances [2, 5]. Main hypothesis of the present study was that these effects can still be detected if tested against a placebo control group in a double-blind experiment. Accordingly, an effect of f = .2 which corresponds to a small to moderate effect following Cohens`classification [28] was used for this study`s power analysis. Furthermore, statistical test-power was set at .95 and significance level (α) was set at .05. Based on recently published results [29] correlation of participants´ testing scores at t_0_ and at t_1_ was estimated at r = .45 for all cognitive tests applied in this study. A 2x4 mixed analysis of variance (mixed ANOVA) including within-subjects factor time-point (t_0_ vs. t_1_) and between-subjects factor treatment (CG vs. LI vs. MI vs. HI) was conducted for data analysis in this study. Therefore, according to our hypothesis, power analysis was conducted for detecting potential interaction effect on cognitive testing performance. It was revealed that 124 participants evenly distributed over the four groups would be required to achieve desired statistical test-power. Test power calculations were conducted using free of charge available statistical test-power computation software GPower 3 [30].

### Participants

The experimental protocol was approved by the ethics committee of the German Sports Science University (Cologne, Germany). One hundred and thirty participants were recruited via announcements of the study in courses at the German Sport Science University and in training sessions of local sports associations. All subjects of the present study participated voluntarily. Students did not receive course credit or comparable advantage for participation. In accordance to the declaration of Helsinki, all participants signed written informed consent prior to participation (S1 and S2).

Subjects were excluded from study participation if they were younger than 18 or older than 35 years of age, had a Body Mass Index (BMI) below 18 or above 30, were pregnant or consumed any illegal drug or prescription medication (other than anti-baby-pill) during previous month. Furthermore, exclusion criteria comprised acute infections, any history of or acute cardiopulmonary, metabolic, neurological or psychiatric diseases, experience with the applied cognitive tests or any limitation in the ability to exercise or complete the cognitive testing procedures. Based on these criteria, 6 subjects were excluded from study participation (3 participants reported a chronic neurological disease or acute neurological symptoms like headache or vertigo, 2 participants reported to suffer from an acute phase of mild depression and 1 participant reported consumption of cannabis during previous month). Furthermore, complete data sets of 3 participants were lost due to technical problems, leading to a total sample size of 121. Detailed descriptive and anthropometric data of tested sample are shown in Table 1.

**Table 1:** Descriptive statistical parameters of central tendency and dispersion for potential confounds separated into treatment groups

	CG		LI		MI		HI	
	n = 31		n = 30		n = 30		n = 30	
	f: 11; m: 20		f: 10; m: 20		f: 7; m: 23		f: 9; m: 21	
	M	SD	M	SD	M	SD	M	SD
**Age (yr)**	23.97	3.37	24.00	3.71	23.37	3.89	23.90	3.73
**Height (m)**	1.77	.09	1.77	.09	1.82	.09	1.78	.09
**Weight (kg)**	69.92	11.84	74.40	13.81	74.20	8.23	70.10	8.39
**BMI (kg/m**^**2**^**)**	22.22	2.43	23.33	1.96	22.51	1.86	21.98	1.75
**MWT-B score (f)**^**+**^	28.23	3.11	28.93	3.50	29.43	3.22	29.07	3.22
**w/Kg**	3.61	.61	3.49	.73	3.64	.54	3.71	.45
**HR (bpm)**	75.13	13.06	73.93	11.15	75.53	14.33	71.43	10.32
**LAC (mmol/l)**^**++**^	1.35	.78	1.11	.26	1.32	.44	1.13	.41
**RT**_**reading**_ **(ms)**	663.74	93.54	658.87	69.21	657.60	67.91	656.50	75.30
**RT**_**naming**_ **(ms)**	624.74	80.91	623.23	68.76	614.50	73.47	616.67	69.49
**Stroop (ms)**	66.87	67.30	72.33	55.88	89.00	59.63	84.33	57.32
**TMT-L A (s)**	16.54	2.52	16.68	3.35	16.10	2.39	16.20	2.80
**TMT-L B (s)**	23.79	3.91	23.10	4.77	21.05	4.38	24.21	5.68
**primacy words (f)**	4.82	1.64	5.00	1.67	5.68	1.57	5.05	1.75
**middle words (f)**	5.77	2.83	5.58	2.60	6.07	3.14	6.08	2.15
**recency words (f)**	3.31	2.14	3.37	1.91	3.63	2.00	3.48	2.02
**words total (f)**	13.90	4.53	13.95	3.73	15.38	4.73	14.60	4.12
	**Mdn**	**Min/Max**	**Mdn**	**Min/Max**	**Mdn**	**Min/Max**	**Mdn**	**Min/Max**
**Education level**^**+++**^	3	3/5	3	3/5	3	3/5	3	3/5
	**frequency**		**frequency**		**frequency**		**frequency**	
**sports student**	22		15		19		18	
**student other**	8		10		6		10	
**PhD-student**	0		1		1		1	
**employed**	1		4		4		1	

CG = control group; LI = low intensity; MI = moderate intensity; HI = high intensity; n = sample size; f = female; m = male; M = Mean; SD = standard deviation; ms = milliseconds; s = seconds; f = frequency; yr = years; cm = centimeter; kg = kilogram; BMI = body mass index; m^2^ = square meter; MWT-B = Multiple Choice Vocabulary Test Version B; w/Kg = watts per kilogram of body weight at graded exercise test on cycle ergometer; bpm = beats per minute; mmol/l = millimoles per liter; Mdn = Median; Min = Minimum; Max = Maximum

^+^ maximum score at MWT-B is 37 points

^++^ for LAC: in MI n = 29 due to data loss of one sample

^+++^1 = no high school diploma, 2 = high school diploma after 10 years of school, 3 = high school diploma after 12 or 13 years of school”, 4 = completed apprenticeship, 5 = university degree

### Potential confounding factors

In the present study, potential confounding factors were supposed to be controlled through randomization. Participants were allocated into treatment groups using an adaptive biased-coin randomization method (urn randomization) [31]. To check if randomization procedure was successful several factors potentially influencing AAE induced cognitive facilitation were measured and tested for imbalances between treatment groups.

### Demographic and anthropometric variables

Demographic variables age, gender, education level and current occupation were determined through self-reporting. Education level was divided into “no high school diploma” (less than 10 years of school education) (coded as 1), “high school diploma after 10 years of school” (coded as 2), “high school diploma after 12 or 13 years of school” (coded as 3), “completed apprenticeship” (coded as 4), “university degree” (coded as 5). Current occupation was divided into “sports-student”, “student of other discipline”, “PhD-student” and “employed person”. Participants`height and weight were measured in the laboratory. Body mass index (BMI) was computed by dividing weight by the square of height (kg/m^2^).

### Intelligence

It was shown that intelligence predicts participants`performances at all cognitive testing procedures applied in the present study [32–34]. Therefore, potentially confounding influence of participants`intelligence was analyzed. Intelligence was captured using the Multiple Choice Vocabulary Test Version B (MWT-B) [35].

### Fitness level

Participants`fitness level was assessed applying a graded exercise test (GXT) on a stationary cycle ergometer (Lode Excalibur Sport, Groningen, Netherlands) until participants reached volitional exhaustion. GXT was applied at least two days prior to actual testing and was conducted according to the following protocol: beginning at 50 watts; increase of 30 watts every three minutes; constant pedaling at 70 rounds per minute (rpm). As measure of physical fitness watts per kilogram of bodyweight (w/Kg) were calculated for each participant for the last completed step at GXT (see Table 1).

### Physical arousal

In the present study, it was hypothesized that physical arousal due to preceding physical activity causes cognitive facilitation. Therefore, potential differences between treatment groups at baseline regarding measures of physical arousal (here: HR and peripheral lactate levels) were investigated.

### Cognitive performances at baseline

Given present study`s design comprising two points of measurement (t_0_ vs. t_1_), it is fundamental to check for potential baseline test score differences between treatment groups before coming to any conclusions about weather exercise affects subsequent cognition.

### Experimental procedure

Data were collected individually within two visits at the Institute of Sports Medicine and Circulation Research of the German Sport Science University. On their first visit to the laboratory, participants were handed out an information sheet containing detailed description of intended study procedures. Participants were given sufficient time to read through the information sheet carefully and they had opportunity to address any questions or concerns about participation. It should be noted that researcher`s hypothesis was neither disclosed to participants in advertisement for study participation nor in information sheet. After participants signed written informed consent, their height and weight were measured. Subsequently, questionnaires concerning demographics, health status, medical history and further exclusion criteria were conducted as interview in order to ensure complete information and quick detection of any exclusion criterion. As far as participants`information did not interfere with above described exclusion criteria, GXT was conducted.

On their second visit to the laboratory, participants were initially interviewed concerning potential changes to their health status. Subsequently, participants completed the MWT-B questionnaire. After participants changed into their sportswear, they completed the baseline assessment (t_0_) comprising the following procedures 20 μl withdrawal of capillary blood from earlobe to determine participants` LAC, HR measurement, cognitive testing. After that, participants assigned to one of the three exercise groups performed a 35 minutes training session on the stationary cycle ergometer while CG participants completed a 35 minutes instructed self-massage intervention using a foam roll (Blackroll, Bottighofen, Swiss). After intervention, participants completed the post-assessment (t_1_) comprising the exact same procedures as at t_0_. It should be noted that cognitive testing was conducted 10 minutes after exercise cessation. To ensure blinding was as effective as possible cognitive testing staff as well as treatment instructors were not aware of the research hypothesis.

### Intervention

Exercise groups (LI, MI and HI) were established on the basis of individual maximum HR (HR_max_) participants had achieved during preceded GXT. Participants assigned to LI exercised at 45–50%, participants assigned to MI at 65–70% and participants assigned to HI at 85–90% of their HR_max_. Exercise session started with a 5 minutes warm up during which participants cycled at 25 watts and were instructed to constantly pedal at 70 rpm. At the end of the warm up interval, wattage was gradually increased by laboratory staff until participants reached their targeted HR range. In order to assure compliance with particular exercise group`s intensity, participants`HR was monitored throughout the whole training session. It was aimed to keep participants within a mid-level of their HR window. Therefore, laboratory staff increased/decreased wattage whenever participants` HR decreased/increased. Participants cycled 30 minutes within the HR range they were assigned to. Exercise interval of 35 minutes was chosen for the following two reasons: (i) it is long enough for most physiological adaptations to physical exertion to run several minutes at a steady state [22]; (ii) it does not induce severe fatigue or dehydration in normal weight healthy young adults; (iii) it keeps participants expenditure of time at reasonable expenses. As CG treatment, a 35-minutes self-massage session using a foam roll guided by an experienced instructor was realized. In order to avoid considerable increase in participants` physical arousal during CG treatment, very low intensity self-massage exercises were selected exclusively. Treatments were carried out by a total of three different persons (student assistants experienced in ergometer and black roll training). Instructors were not restricted to one specific treatment but carried out all four treatments approximately in equal parts (as can be seen from supplemental data set 1)

### Cognitive assessments

All tests applied in this study were computer based. Stroop task and Langensteinbacher version of the TMT (TMT-L) [36] were operated via computerized testing platform Wiener Test-System (WTS) (Schuhfried, Vienna, Austria) while free recall task was applied using Microsoft PowerPoint. In order not to be compelled to switch between different computer programs and thereby to avoid distracting participants, Stroop task and TMT-L were always administered preceding free recall task. Thus, only order of Stroop task and TMT-L was counterbalanced across participants while free recall was always administered last. Cognitive assessment in this study exclusively comprised Stroop task, TMT and free recall task. Duration of cognitive testing at each time-point (t_0_ and t_1_) was approximately 20 minutes. Cognitive testing was carried out by a total of four different persons (student assistants experienced with the here applied testing software). Test supervisors are roughly distributed to the four experimental conditions in equal parts.

#### Stroop task

In the present study, the German version of the computerized Stroop task from the WTS was applied [37]. This Stroop task uses four different colors (red, blue, green and yellow) and comprises the following conditions: reading color words displayed in black font color (reading baseline), naming ink color of differently colored rectangles (naming baseline), naming ink color of color words in incongruent format (Stroop condition). Conditions are tested in separate consecutive testing blocks (order as above). Each testing block comprises 128 items which are presented in the center of the upper third of the screen one after another. Participants react using a special keyboard which comprises four correspondingly colored buttons. Before actual testing a training period is conducted comprising 25 items. However, actual testing only starts if testing software detects rule comprehension and habituation. Otherwise, practice trial is expanded.

WTS Stroop testing software provides the medians of participants` reaction time (RT) at reading baseline (RT_reading_) and at naming baseline condition (RT_naming_). Furthermore, it computes a Stroop effect variable (Stroop) as measure of interference control by subtracting RT_naming_ from median RT at Stroop condition. All those 3 variables were included into further analyses. Manual of the here applied Stroop task reports Cronbach`s alpha values for provided variables between .93 and .99 [37] indicating excellent internal consistency.

#### Trail Making Test

In the present study, two parallel versions of the TMT-L in counterbalanced order operated via WTS (Schuhfried, Vienna, Austria) were applied. TMT-L contains all features of former paper-pencil versions of the TMT [38]. In TMT-L part A, targets are randomly distributed numbers from 1 to 25 which participants are supposed to connect as quickly and correctly as possible in ascending order. In TMT-L part B, targets are randomly distributed numbers (1 to 13) and letters (A to L) which participants are supposed to connect as quickly and correctly as possible in ascending order alternating between numbers and letters. Each mistake is immediately pointed out by the software through an acoustic signal and must be instantly corrected before proceeding. Before actual testing a training period is conducted comprising example problems with 9 numbers (part A) and 5 numbers/4 letter (part B), respectively. However, actual testing only starts if testing software detects rule comprehension and habituation. Otherwise, practice trial is expanded.

The time participants require completing TMT part A had been described as a valid measure of selective attention [39] while the time participants require completing TMT part B had been described as valid measure of executive functioning subdomain set-shifting [39]. TMT-L testing software provides participants`time needed for completing part A (TMT-L A) and participants`time needed for completing part B (TMT-L B). Both those variables were used for further analyses. TMT-L testing manual reports a greatest lower bound of .95 for TMT-L A and of .84 for TMT-L B [36] indicating good to excellent reliability.

#### Free recall task

For the free recall task of the present study two 40-item word lists were created as parallel forms (see procedure at Behrendt [40]). Application of lists at t_0_ and at t_1_ was counterbalanced across participants. For each participant and each list, words were presented in random order. Participants started testing by pressing the left mouse key. Before the first word, an attentional cue (“attention”) was presented in the center of the screen for 4 seconds. After that, words were presented one after another. Each word was presented individually in 48-point black colored Arial font in the center of the screen against white background for 4 seconds. Following the last word a 90 seconds consolidation period was provided during which participants were allowed but not specifically instructed to rehearse the list (compare to procedure at Coles & Tomporowski [12]). Then, a sentence on the screen instructed participants to verbally recall as many words from the list, in any order, using a built in computer microphone that recorded participants recall. When participants did not recall a word for longer than 20 seconds they were asked by the test instructor if they could recall any more items. If they did not recall anymore items for another 10 seconds recall period was ended. Words were considered correct if they were present in the to-be remembered list, with minor pronunciation errors and plural-singular substitutions ignored. Variables collected and used for further analyzing in the present study were number of words recalled from primacy portion (first 10 words of the list) (primacy words), number of words recalled from the middle section (middle words) and number of words recalled from recency portion (last 10 words of the list) (recency words). Furthermore, total number of words recalled from the list (words total) was captured and analyzed as comprehensive measure of declarative long-term memory performance. Recordings of participants`free recall performances were evaluated by two independent, blinded investigators. In case of differing evaluation between investigators, the mean of both ratings was calculated and used for further analysis.

### Data analysis

Potential baseline differences between treatment groups in age, height, weight, BMI, MWT-B scores, w/Kg and cognitive testing scores were investigated using separate one-way analyses of variance (ANOVAs). Basic parametric statistical models`assumption of normality was not further investigated. Due to central limit theorem, we assumed approximate normality of sampling distribution. Moreover, ANOVA was shown to be robust to violations of normality assumption as long as groups sizes are (fairly) equal (for overview see Field [41]). ANOVA assumption of homogenous variances for between-subjects factor levels was tested using Levene test and, in case of inhomogeneous variances, F-test was adjusted using Brown-Forsythe F (F_BF_). Partial eta-square (η^2^_p_) values are reported as effect size estimates. Potential baseline difference between treatment groups regarding education level were analyzed using separate Kruskal-Wallis test. Potential baseline differences regarding distribution of gender and current occupation were investigated using separate Fischer`s exact tests.

As measure of the extent of absolute agreement between the two independent, blinded investigators evaluating the recordings of participants` free recall performances interclass correlation coefficients (ICC) as well as their confidence intervals (reported in square brackets) were calculated. To determine the effect of treatment on HR, LAC and cognitive testing outcomes separate 2x4 mixed ANOVAs were conducted. For the above reasons, assumption of normality within each cell was not investigated. Again, ANOVA assumption of homogenous variances for between-group factor levels was tested using Levene test and, in case of inhomogeneous variances, F-test was adjusted using Brown-Forsythe F (F_BF_). η^2^_p_ values are reported as effect size estimates. If 2x4 mixed ANOVA revealed a significant time-point*treatment interaction effect on any variable, this effect was further investigated carrying out Bonferroni corrected post hoc pairwise comparisons of baseline-post-assessment difference values (Δ-values). Δ-values were always calculated by subtracting baseline values from post assessment values. Furthermore, in case of significant interaction effect, the course of Δ-value means from CG to HI was investigated using trend analysis (polynomial contrasts).

To explore potential association between LAC and cognitive performances at t_1_ Pearson´s bivariate correlation coefficients were calculated separately for each treatment group and tested for significance using general linear model. Assumption of normality had not been tested for above mentioned reasons. Alpha error accumulation was controlled through Benjamini-Hochberg procedure [42]. Due to the explorative nature of the correlational analyses a quite high false discovery rate was chosen (Q = .2).

For all inferential statistical analyses, significance was defined as p-value less than .05. All descriptive and inferential statistical analyses were conducted using SPSS 22® (IBM®, Armonk, NY, USA).Two-tailed probability tests were used throughout all inferential statistical testing.

## Results

### Post hoc power analyses

Statistical test power for detection of a time-point*treatment interaction effect on cognitive performance at a 2x4 mixed ANOVA was analyzed separately for each cognitive testing outcome. Post hoc power analyses were conducted for decreasing effect sizes (f = .2; f = .15; f = .1) with significance level set at .05, achieved sample size of N = 121 and empirical correlations between specific test scores at t_0_ and at t_1_ (see Table 2) considered. It was revealed that an effect of f = .2, provided this effect really does exist on the population level, would have been detected in the present study with a statistical test power of .82 to .99. Even reducing the relevant effect size to f = .15, statistical test power stayed above common limit value of .8 [39] for all cognitive testing variables but TMT-L B (1-β = .78), primacy words (1-β = .55), middle words (1-β = .55) and recency words (1-β = .6). Further reducing relevant effect size to f = .1, however, decreased statistical test power below .8 for all cognitive testing variables but RT_reading_ (1-β = .91). Detailed results of post hoc power analyses are displayed in Table 2.

**Table 2:** Key components and results of post hoc power analyses.

	N	r	1-β at f = .2	1-β at f = .15	1-β at f = .1
**RT**_**reading**_	121	.766	.999	.987	.761
**RT**_**naming**_	121	.838	.999	.999	.906
**Stroop**	121	.518	.971	.801	.429
**TMT-L A**	121	.575	.985	.853	.481
**TMT-L B**	121	.497	.965	.782	.413
**primacy words**	121	.175	.817	.546	.261
**middle words**	121	.483	.96	.77	.402
**recency words**	121	.261	.861	.598	.289
**words total**	121	.618	.992	.891	.528

N = sample size; r = Correlation among repeated measures; 1-β = post hoc test power; f = effect size measure; RT_reading_ = participants`reaction time reading color words displayed in black font color; RT_naming_ = participants`reaction time naming ink color of differently colored rectangles; Stroop = RT_naming_ subtracted from participants`reaction time naming ink color of words at same time semantically expressing different colors; TMT-L A = Trail Making Test Langensteinbacher version part A; TMT-L B = Trail Making Test Langensteinbacher version part B

### Potential confounders

Separately conducted one-way ANOVAs revealed no significant differences among treatment groups regarding age, height, weight, BMI, MWT-B scores, w/KG, HR, LAC as well as free recall, Stroop and TMT-L results at t_0_ (F(3, 117) = .052–2.649, p = .052–.984, η^2^_p_ = .001–.064) (for weight and w/KG violation of assumption of homogenous variances was detected through significant Levene test. Therefore, F-test was adjusted using Brown-Forsythe F for these variables). However, for participants`BMI (p = .061) and TMT-L B score (p = .052) at baseline nonsignificant trends were found. Concerning participants`education level as well as distribution of gender and current occupation, treatment groups also did not deviate significantly from each other as was shown through Kruskal-Wallis test (H(3) = 3.8, p = .2) and Fischer`s exact tests, respectively (p = .559–.767). Descriptive statistical parameters of central tendency and dispersion for potential confounds separated into treatment groups are presented in Table 1.

### Manipulation check

**Fig 1:**
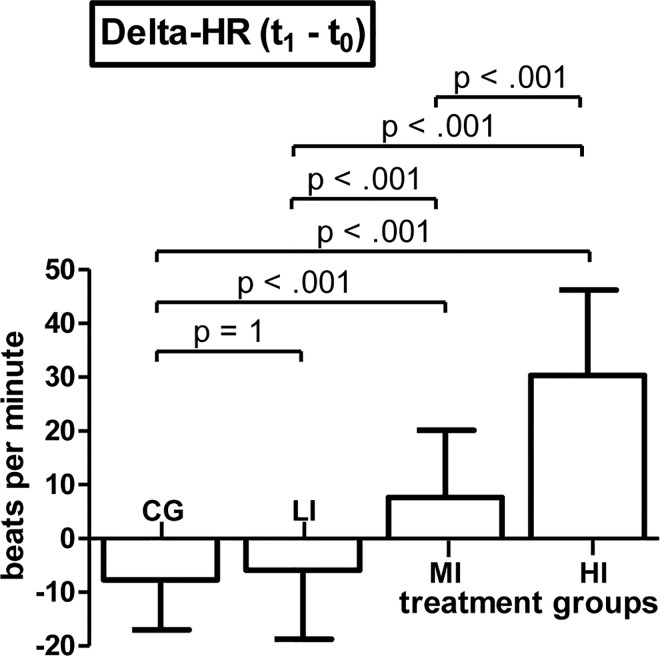
Results of the manipulation check. Means and SD of pre to post treatment difference values of participants heart rate (Delta-HR) subdivided by treatment groups (CG = control group (n = 31), LI = low intensity group (n = 30), MI = moderate intensity group (n = 30), HI = high intensity group (n = 30))

2x4 mixed ANOVA on HR revealed a significant time-point*treatment interaction effect (F(3, 117) = 56.646, p < .001, η^2^_p_ = .592), a significant main effect of within-subjects factor time-point (F(1, 117) = 27.108, p < .001, η^2^_p_ = .188), a significant main effect of between-subjects factor treatment (F(3, 117) = 15.569, p < .001, η^2^_p_ = .285). Post hoc comparisons of treatment groups` Δ-HR values revealed no significant difference between CG and LI (p = 1, d = .159) while MI showed significantly higher mean Δ-HR compared to CG (p < .001, d = -1.395) and also compared to LI (p < .001, d = 1.07). HI showed a significantly higher mean Δ-HR than CG (p < .001, d = -2.932), LI (p < .001, d = 2.509) and MI (p < .001, d = -1.587) (Fig 1). Trend analysis of treatment groups`Δ-HR values revealed that the course of increase in mean Δ-HR values from CG to HI significantly fits a linear (F(1, 117) = 150.915, p < .001) as well as a quadratic trend (F(1, 117) = 20.17, p < .001).

### Cognitive assessments

#### Stroop task

2x4 mixed ANOVA on RT_reading_ revealed a significant time-point*treatment interaction effect (F(3, 117) = 3.075, p = .03, η^2^_p_ = .073). Main effect of within-subjects factor time-point (F(1, 117) = 164.872, p < .001, η^2^_p_ = .694) also reached statistical significance while main effect of between-subjects factor treatment (F(3, 117) = .613, p = .608, η^2^_p_ = .015) did not. Post hoc comparisons and trend analyses of treatment groups` Δ-RT_reading_ values detected significant difference only between LI and HI (p = .034, d = .784). All other groups showed no significant differences between each other (p = .123–1, d = .132–.693). The course of Δ-RT_reading_ from CG to HI significantly fits a linear (F(1, 117) = 4.268, p = .041) but not a quadratic trend (F(1, 117) = 1.822, p = .18).

**Fig 2:**
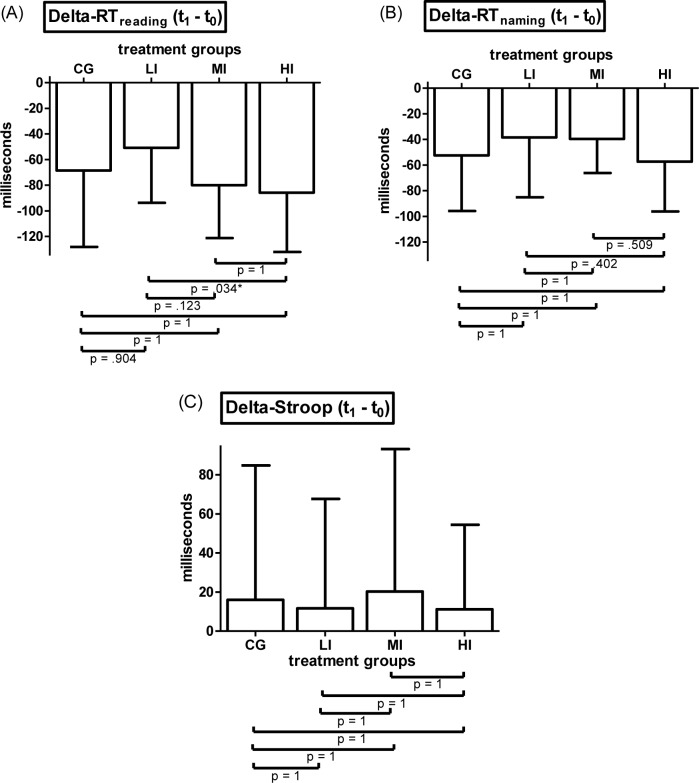
Results of the Stroop task. (A) Means and SD of baseline to post-assessment difference values of participants`reaction times at Stroop condition reading color words (Delta-RT_reading_) subdivided by treatment groups. (B) Means and SD of baseline to post-assessment difference values of participants`reaction times at Stroop condition naming ink color of differently colored rectangles (Delta-RT_naming_) subdivided by treatment groups. (C) Means and SD of baseline to post-assessment difference values of participants`reaction times at Stroop condition naming ink color of color words in incongruent format (Delta-Stroop). (CG = control group (n = 31), LI = low intensity group (n = 30), MI = moderate intensity group (n = 30), HI = high intensity group (n = 30), pairwise comparisons are Bonferroni corrected)

Results of separately conducted 2x4 ANOVAs on RT_naming_ and Stroop revealed an identical pattern in terms of statistical significance of investigated effects. For both variables the effect of time-point-treatment interaction did not reach statistical significance (RT_naming_: F(3, 117) = 1.694, p = .172, η^2^_p_ = .042; Stroop: F(3, 117 = = .144, p = .933, η^2^_p_ = .004). Main effect of factor time-point was significant (RT_naming_: F(1, 117) = 169.77, p < .001, η^2^_p_ = .592; Stroop: F(1, 117 = = 7.057, p = .009, η^2^_p_ = .057). Main effect of factor treatment was not significant (RT_naming_: F(3, 117) = .302, p = .824, η^2^_p_ = .008; Stroop: F(3, 117 = = 1.309, p = .275, η^2^_p_ = .032) Means and SD of Δ-RT_reading_, Δ-RT_naming_ and Δ-Stroop subdivided by intervention groups are displayed in Fig 2 (Bonferroni corrected pairwise comparisons are included in figures).

#### TMT-L

**Fig 3:**
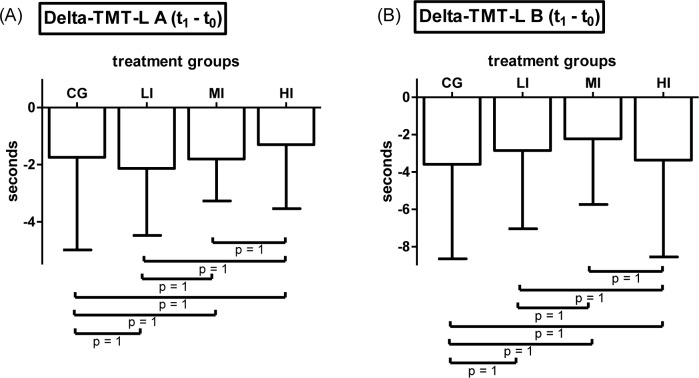
Results of the TMT-L. (A) Means and SD of baseline to post-assessment difference values of participants`duration to complete part A of the Langensteinbacher version of the Trail Making Test—(Delta-TMT-L A) subdivided by treatment groups. (B) Means and SD of baseline to post-assessment difference values of participants`duration to complete part B of the Langensteinbacher version of the Trail Making Test—(Delta-TMT-L B) (CG = control group (n = 31), LI = low intensity group (n = 30), MI = moderate intensity group (n = 30), HI = high intensity group (n = 30) pairwise comparisons are Bonferroni corrected)

For both variables, TMT-L A and TMT-L B, separately conducted 2x4 mixed ANOVAs did not reveal a significant time-point*treatment interaction effect (TMT-L A: F(3, 117) = .602, p = .615, η^2^_p_ = .015; TMT-L B: F(3, 117) = .538, p = .657, η^2^_p_ = .014). Main effect of within-subjects factor time-point showed statistical significance for both those variables (TMT-L A: F(1, 117) = 63.028, p < .001, η^2^_p_ = .35; TMT-L B: F(1, 117) = 52.826, p < .001, η^2^_p_ = .311). Main effect of between-subjects factor treatment did not reach statistical significance for TMT-L A and TMT-L B (TMT-L A: F(3, 117) = .251, p = .86, η^2^_p_ = .006; TMT-L B: F(3, 117) = 2.629, p = .053, η^2^_p_ = .063) Means and SD of Δ-TMT-L A and Δ-TMT-L B as a function of treatment are shown in Fig 3 (Bonferroni corrected pairwise comparisons are included in figures).

#### Free recall

ICC of free recall data ratings revealed high accordance between both raters for all free recall variables at both time points (ICC = .991–.998 [.988–.997, .990–.998]). Conducted 2x4 mixed ANOVA on primacy words did not reveal a significant time-point*treatment interaction effect on primacy words (F(3, 117) = 1.793, p = .152, η^2^_p_ = .044). Main effect for factor time-point was significant (F1, 117) = 5.544, p = .02, η^2^_p_ = .045) while main effect of factor treatment was not (F(3, 117) = 1.733, p = .164, η^2^_p_ = .043).

**Fig 4:**
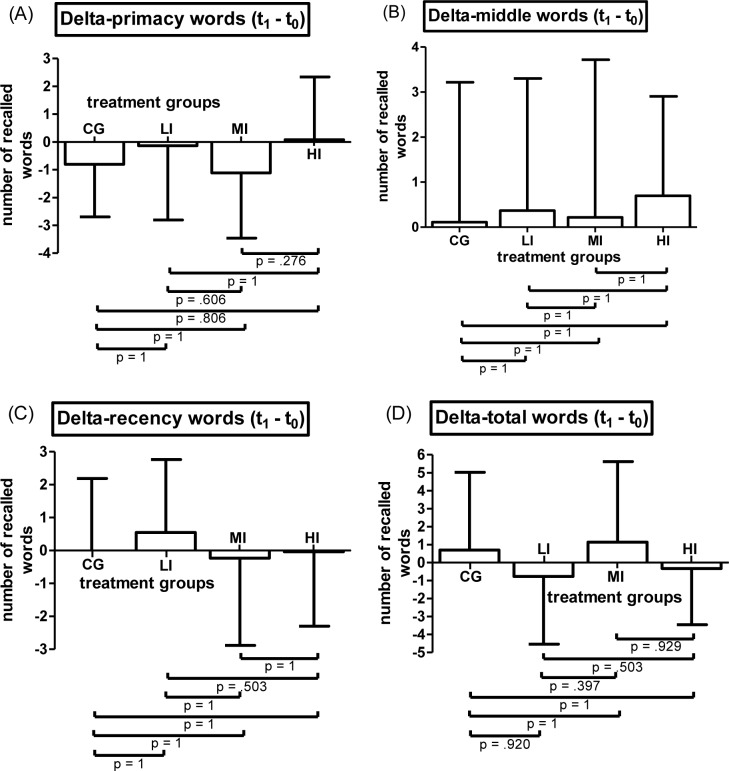
Results of the Free recall. (A) Means and SD of baseline to post-assessment difference values of participants`number of words recalled from primacy portion (first 10 words of the list) (Delta-primacy words) subdivided by treatment groups. (B) Means and SD of baseline to post-assessment difference values of participants`number of words recalled from the middle section (the middle 20 words) (Delta-middle section) subdivided by treatment groups. (C) Means and SD of baseline to post-assessment difference values of participants`number of words recalled from the recency portion (last 10 words of the list) (Delta-recency words) subdivided by treatment groups. (D) Means and SD of baseline to post-assessment difference values of participants`number of words recalled from the list (Delta-words total) subdivided by treatment groups(CG = control group (n = 31), LI = low intensity group (n = 30), MI = moderate intensity group (n = 30), HI = high intensity group (n = 30) pairwise comparisons are Bonferroni corrected)

In terms of middle words, recency words and total words, separately conducted 2x4 mixed ANOVAs neither detected a significant interaction effect (middle words: F(3, 117) = .244, p = .879, η^2^_p_ = .006; recency words: F(3, 117) = 1.116, p = .345, η^2^_p_ = .028; total words: F(3, 117) = 1.488, p = .221, η^2^_p_ = .037) nor a significant main effect of within-subjects factor time-point (middle words: (F1, 117) = 1.666, p = .199, η^2^_p_ = .014; recency words: (F1, 117) = .033, p = .855, η^2^_p_ = 0; total words: (F1, 117) = .254, p = .615, η^2^_p_ = .002) nor a significant main effect of between-subjects factor treatment (middle words: F(3, 117) = .469, p = .704, η^2^_p_ = .012; recency words: F(3, 117) = .458, p = .712, η^2^_p_ = .012; total words: F(3, 117) = .62, p = .603, η^2^_p_ = .016). Means and SD of Δ-primacy words, Δ-middle words, Δ-recency words and Δ-total words subdivided by intervention group are displayed in Fig 4 (Bonferroni corrected pairwise comparisons are included in figures).

#### Associations between LAC and cognitive performances

Empirical associations between LAC and cognitive performances at t_1_ were rather small (r = -.392–.338). Even though a fairly high false discovery rate was applied (Q = .2), none of these associations reached statistical significance when controlling alpha error accumulation through Benjamini-Hochberg procedure. Detailed results of correlation analysis are displayed in Table 3.

**Table 3:** Associations between LAC and cognitive testing performances at t_1._

		r	p	Rank (i)	(i/m)Q
**CGn = 31**	**RT**_**reading**_ **(sec)**	-.169	.364	16	0.089
	**RT**_**naming**_ **(sec)**	-.086	.646	30	0.167
	**Stroop (sec)**	-.151	.419	19	0.106
	**TMT-L A (sec)**	-.11	.554	26	0.144
	**TMT-L B (sec)**	-.106	.571	28	0.156
	**primacy words (f)**	.082	.659	31	0.172
	**middle words (f)**	-.172	.356	15	0.083
	**recency words (f)**	-.28	.128	3	0.017
	**words total (f)**	-.178	.339	13	0.072
**LIn = 30**	**RT**_**reading**_ **(sec)**	.038	.84	36	0.200
	**RT**_**naming**_ **(sec)**	-.145	.444	20	0.111
	**Stroop (sec)**	.047	.805	34	0.189
	**TMT-L A (sec)**	.247	.189	8	0.044
	**TMT-L B (sec)**	.215	.253	10	0.056
	**primacy words (f)**	-.392	.032	1	0.006
	**middle words (f)**	.126	.507	25	0.139
	**recency words (f)**	-.206	.275	11	0.061
	**words total (f)**	-.142	.455	21	0.117
**MIn = 30**	**RT**_**reading**_ **(sec)**	.132	.493	24	0.133
	**RT**_**naming**_ **(sec)**	.175	.346	14	0.078
	**Stroop (sec)**	.338	.073	2	0.011
	**TMT-L A (sec)**	.113	.558	27	0.150
	**TMT-L B (sec)**	.107	.581	29	0.161
	**primacy words (f)**	.289	.129	4	0.022
	**middle words (f)**	-.158	.412	18	0.100
	**recency words (f)**	-.054	.782	33	0.183
	**words total (f)**	-.045	.819	35	0.194
**HIn = 30**	**RT**_**reading**_ **(sec)**	.24	.202	9	0.050
	**RT**_**naming**_ **(sec)**	.17	.37	17	0.094
	**Stroop (sec)**	-.075	.692	32	0.178
	**TMT-L A (sec)**	.136	.474	23	0.128
	**TMT-L B (sec)**	.196	.299	12	0.067
	**primacy words (f)**	-.253	.178	7	0.039
	**middle words (f)**	-.139	.464	22	0.122
	**recency words (f)**	-.267	.154	6	0.033
	**words total (f)**	-.283	.130	5	0.028

LAC = whole blood lactate concentration; t_1_ = assessment after intervention, Rank = rank of p-values ordered from smallest to largest; m = number of tests; (i/m)Q = Benjamini-Hochberg critical value; CG = control group, LI = low intensity group, MI = moderate intensity group, HI = high intensity group

## Discussion

Acute exercise induced cognitive facilitation is widely understood as a result of physiological adaptations to the preceding physical exertion [1, 5–7]. However, published studies on the effects of AAE on subsequent cognition in healthy young adults do not allow such a conclusion since they did not control for placebo, Hawthorne and subject expectancy effects. Against this backdrop, primary aim of the present study was to apply a research design that, at a high degree of validity, allows attributing effects of AAE on cognition to the physiological impact of the preceding exertion and therefore provide robust evidence for a physiological basis of exercise induced cognitive facilitation. Furthermore, an intensity-dependent AAE induced cognitive facilitation and a mediating role of peripheral lactate was hypothesized.

Different to what was expected, reported AAE induced cognitive facilitation in healthy young adults could not be replicated in the present study. None of the here applied AAE intensities did significantly improve subsequent cognitive testing performances if compared to instructed self-myofascial release training control group. Present study`s power analysis allows concluding that the here applied exercise regimes have no effect of f = .2 or larger on subsequent Stroop, TMT-L or free recall performances if compared to a not physically arousing foam roll self-massage intervention taking into account a risk of type II error (β) ≤.183. Accordingly, one might argue that AAE induced cognitive facilitation is not due to physiological processes induced by preceding exercise. However, before one draws this conclusion a closer look should be taken on some critical aspects of the present study.

Chang and colleagues [5] discovered in their meta-analysis that cognitive test administration 11 to 20 minutes after cessation of exercise generally results in the biggest effects and that these effects subside following a longer (>20 min) delay. In the present study, cognitive testing was conducted 10 minutes after exercise and TMT and Stroop task were always administered before free recall task. Duration of TMT and Stroop task was about 10 minutes falling right into the window of the greatest expected effects. However, free recall task was administered at a delay of about 20 minutes after exercise cessation potentially explaining why exercise did not exert a positive effect on participants`performance at that task.

It remains unclear how comparable exercise and control treatment were in terms of biasing psychosocial stimuli. It cannot be ruled out that instructed self-massage foam roll intervention has raised much higher expectations of positive effects on subsequent cognitive performances and, accordingly, had been more biased by placebo effect than exercise intervention. This argument becomes all the more important since recently there is a trend to investigate and promote the effect of mindfulness inducing activities like meditation and yoga on subsequent cognition [43]. Possibly, participants interpreted self-massage foam roll intervention as such a mindfulness inducing activity and, accordingly, developed positive expectation of cognitive benefits. In a recently published study Stothart and colleagues [44] showed that a non-aerobic training intervention comprising yoga, balance and stretching exercises (3x/week for 6 months) raised higher expectations regarding cognitive benefits in elderly than an aerobic training intervention (3x/week for 6 months). However, this study by Stothart et al. looked for expectations of training and not acute exercise induced benefits in elderly and not in young healthy adults, it did not list testing procedure applied here and, even more important, looked for expectations of different exercise contents to facilitate cognition.

Furthermore, it cannot be ruled out that participants during instructed self-massage foam roll intervention gained more attention than participants during exercise treatments and therefore might have become more prone to Hawthorne effect related bias. Similarly, one might argue that instructed self-massage foam roll intervention required a higher level of attention and alertness from participants than what exercising on a cycle ergometer did and that these differences affected subsequent cognitive testing performances. However, despite potential advantages for control group regarding psychosocial stimuli that might have made up for potential physiological benefits of the exercise treatments our results indicate that AAE induced cognitive facilitation is not exclusively based on physiological effects. Other psychosocial aspects seem to play an equal role. Therefore, future studies should further investigate potential physiological basis of exercise induced cognitive facilitation but should also explicitly investigate psychosocial factors like expectations, social attention and psychological arousal. In this context, psychological arousal theories might be applied as theoretical framework. Optimizing exercise interventions not only in terms of physiological responses but also in terms of an ideal psychosocial environment might help physical activity become a potential optimization tool for subsequent cognitive challenges in academic contexts and professional fields (e.g. aviation and military) as has recently been proposed [13].

Last but not least, participants might not have been sufficiently habituated to cognitive testing procedures with the preceding training period and, therefore, changes from t_0_ to t_1_ might reflect learning instead of actual improvement of the critical cognitive performance. That issue has not yet been properly addressed in the acute exercise–cognition literature. Learning could include e.g. motoric aspects of operating the computer test device. Learning effects are a problem since applied treatments might induce different effects on learning making it a confounding factor. It was shown, however, that exercise improves learning [3]. Accordingly, it seems more likely that confounding due to learning was in favor of the here applied AAE regimes. Still, future studies should familiarize participants to the applied cognitive testing procedures to eliminate potential confounding by learning and provide data showing that habitation was successful.

In the present study, peripheral lactate could not be confirmed as mediator of exercise induced cognitive facilitation. A significant positive association between peripheral lactate and cognitive testing performances could not be shown for any treatment group and any cognitive testing variable. Possibly, the duration between elevated peripheral lactate levels during treatment and subsequent cognitive testing was too short. Peripheral lactate is transported actively across the blood brain barrier (BBB) through monocarboxylate transporter (MCT) 1 [45]. This process and the uptake of lactate from the intercellular space into neurons take time. Peripheral lactate might, therefore, become a more effective mediator of AAE induced cognitive facilitation if the cognitive challenges come with a longer break after the acute exercise. Future studies should investigate the role of peripheral lactate in sustained effects of exercise on subsequent cognition.

## Conclusions

In conclusion, the present study raises doubts that AAE induced cognitive facilitation is (exclusively) based on physiological effects. Even if there is a substantial contribution of physiological adaptations to AAE in reported AAE induced cognitive facilitation, in this study, peripheral lactate could not be confirmed as such a factor. Our results suggest that other psychosocial aspects like expectations and social attention play an important role in AAE induced cognitive facilitation.

## Supporting Information

S1 File Written informed consent (German). (DOCX)

S2 File Written informed consent (English). (DOCX)

S1 Table Whole data set without free recall. (XLS)

S2 Table Free recall data. (XLS)
