# Supplementary material for: Do Reported Effects of Acute Aerobic Exercise on Subsequent Higher Cognitive Performances Remain if Tested against an Instructed Self-Myofascial Release Training Control Group? A Randomized Controlled Trial
Source: PLoS One. 2016 Dec 8;11(12):e0167818. doi: 10.1371/journal.pone.0167818 (PMC5145178; doi:10.1371/journal.pone.0167818)
Supplement: S1 File — (DOCX) [file pone.0167818.s001.docx]

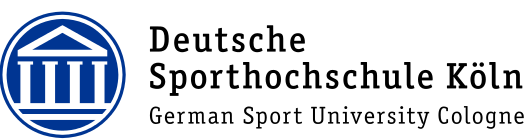
**Institut für Kreislaufforschung und Sportmedizin**

**Zustimmung zur Teilnahme**

- Diese Studie wird im Rahmen eines Forschungsprojektes des Instituts für Kreislaufforschung und Sportmedizin der Deutschen Sporthochschule durchgeführt. Unter der Supervision von Prof. Dr. W. Bloch sind Dipl. Sportwiss B.Sc Max Oberste ([oberstemax@hotmail.com](mailto:oberstemax@hotmail.com)) und Dipl. Sportwiss B.Sc. Neurowiss. Phillip Zimmer ([p.zimmer@dshs-koeln.de](mailto:p.zimmer@dshs-koeln.de)) für die Studienleitung verantwortlich und erste Ansprechpartner für ihre Rückfragen.
- Die Untersuchungen werden im Rahmen von zwei Besuchen im Institut für Kreislaufforschung und Sportmedizin durchgeführt. Während Ihres ersten Besuchs werden demographische und anthropometrische Daten erfasst. Außerdem wird Ihre körperliche Fitness getestet. Dafür absolvieren Sie einen Stufentest auf einem Fahrradergometer wobei Ihnen auch kapillares Blut aus Ihrem Ohrläppchen zur Laktatbestimmung entnommen wird. Während Ihres zweiten Besuchs werden Sie eine etwa 30 minütige Trainingseinheit absolvieren. Vor und nach dieser Trainingseinheit werden Sie gebeten, eine kognitive Testbatterie zu bearbeiten. Vor und nach diesen Tests wird Ihnen ebenfalls kapillares Blut aus Ihrem Ohrläppchen zur Laktatdiagnostik entnommen.

• Alle im Rahmen der Untersuchung gesammelten Daten werden anonymisiert und nur für Forschungszwecke weiterverarbeitet. Die Mitarbeiter der Untersuchung sind zur Verschwiegenheit verpflichtet. Nicht mehr benötigte Daten werden unverzüglich gelöscht. Andere Wissenschaftler oder Mitglieder der Ethikkommissionen können u.U. in Daten der Untersuchung Einsicht nehmen. Dies geschieht jedoch ebenfalls unter strikter Einhaltung der Vertraulichkeit.

- Die Teilnahme an der Untersuchung ist freiwillig. Sie können jederzeit und ohne Angabe von Gründen ihre Zustimmung zur Teilnahme widerrufen, ohne dass Ihnen deswegen Nachteile entstehen.
- Sie können detaillierte Informationen über die Studien erhalten, sobald die Datenerhebung vollständig abgeschlossen ist. Wenn Sie dies wünschen, tragen Sie bitte unten in dem dafür vorgesehenen Feld Ihre E-Mail-Adresse ein.

*Ich habe die oben angeführten Informationen über das Forschungsprojekt gelesen und verstanden. Mir wurde ausreichend Zeit gegeben, Fragen über das Projekt und meine Teilnahme zu stellen. Ich nehme freiwillig an dieser Studie teil. Ich verstehe, dass ich jederzeit und ohne Angabe von Gründen oder irgendwelche resultierenden Nachteile meine Studienteilnahme zurückziehen kann. Das Procedere bezüglich der Geheimhaltung von Daten wurde mir erklärt. Die Verwendung der Daten im Rahmen dieses Projektes wurde mir erklärt. Ich verstehe, dass andere Wissenschaftler gegebenenfalls Einsicht in die Daten nehmen können, wenn sie sich zur Geheimhaltung verpflichten und zu den hier dargelegten Richtlinien entsprechen.*

Datum____________________ Unterschrift:_____________________

E-Mail-Adresse:___________________________________________________

(Bitte angeben, falls Sie über die Ergebnisse der Studie informiert werden wollen sobald die Erhebung abgeschlossen ist)
