# Supplementary material for: Do Reported Effects of Acute Aerobic Exercise on Subsequent Higher Cognitive Performances Remain if Tested against an Instructed Self-Myofascial Release Training Control Group? A Randomized Controlled Trial
Source: PLoS One. 2016 Dec 8;11(12):e0167818. doi: 10.1371/journal.pone.0167818 (PMC5145178; doi:10.1371/journal.pone.0167818)
Supplement: S2 File — (DOCX) [file pone.0167818.s002.docx]

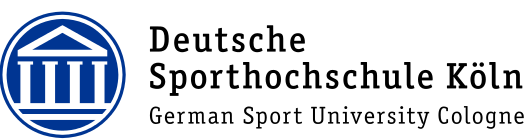
**Institut für Kreislaufforschung und Sportmedizin**

**Informed Consent**

- This study is conducted as part of a research project of the Institute of Sports Medicine and Circulation Research of the German Sport Science University. Under the supervision of Prof. Dr. Bloch Dipl. Sports Scientist B.Sc. Max Oberste ([oberstemax@hotmail.com](mailto:oberstemax@hotmail.com)) and Dipl. Sport Scientist BSc. Philipp Zimmer ([p.zimmer@dshs-koeln.de](mailto:p.zimmer@dshs-koeln.de)) are responsible for this study and main contact for any of your questions.
- The examinations will be conducted within two visits at the Institute of Sports Medicine and Circulation Research. On your first visit, demographic and anthropometric data will be collected and your physical fitness will be tested. For that purpose, you will complete a graded exercise test on a cycle ergometer where capillary blood will be withdrawn from your earlobe to determine your peripheral lactate levels. On your second visit you will complete an approximately 30 minutes training session. Before and after that training session you will be asked to complete a cognitive testing battery. Before and after cognitive testing, capillary blood from your earlobe will be withdrawn again.
- All data that will be collected in this study will be anonymized und only further analyzed for research purposes. Research staff members are sworn to secrecy. Data that is of no use for further scientific analyzation will be erased promptly. Other researchers or members of the ethics committee might look at some of the data. However, this will also happen strictly maintaining confidentiality.
- Participation in this study is voluntarily. At all times and without giving reasons you can revoke your approval of participation. Doing so will cause you no disadvantages.
- You can receive detailed information about this study as soon as data collection is completed. If you wish so, please state your email in the below provided field.

*I have read and understood the above provided information about the research project. I have been given the opportunity to ask questions about the project and my participation. I voluntarily agree to participate in the project. I understand I can withdraw at any time without giving reasons and that I will not be penalized for withdrawing nor will be questioned why I have withdrawn. The procedures regarding confidentiality have been clearly explained to me. The use of the data in research has been explained to me. I understand that other researchers will have access to this data if they agree to preserve the confidentiality of the data and if they agree to the term specified in this form*

Date____________________ Sign_____________________

E-Mail:___________________________________________________

(Please state if you wish to receive detailed information about this study as soon as data collection is completed)
